# Not so biodegradable: Polylactic acid and cellulose/plastic blend textiles lack fast biodegradation in marine waters

**DOI:** 10.1371/journal.pone.0284681

**Published:** 2023-05-24

**Authors:** Sarah-Jeanne Royer, Francesco Greco, Michaela Kogler, Dimitri D. Deheyn

**Affiliations:** 1 Marine Biology Research Division, Scripps Institution of Oceanography, UC San Diego, La Jolla, CA, United States of America; 2 Center for Marine Debris Research, Hawaii Pacific University, Waimanalo, HI, United States of America; 3 Shaanxi Key Laboratory of Early Life & Environments and Department of Geology, State Key Laboratory of Continental Dynamics, Northwest University, 710069, Xi’an, China; 4 Department of Plant and Environmental Sciences, Weizmann Institute of Science, Rehovot, Israel; 5 Lenzing AG, Lenzing, Austria; University of Queensland, AUSTRALIA

## Abstract

The resistance of plastic textiles to environmental degradation is of major concern as large portions of these materials reach the ocean. There, they persist for undefined amounts of time, possibly causing harm and toxicity to marine ecosystems. As a solution to this problem, many compostable and so-called biodegradable materials have been developed. However, to undergo rapid biodegradation, most compostable plastics require specific conditions that are achieved only in industrial settings. Thus, industrially compostable plastics might persist as pollutants under natural conditions. In this work, we tested the biodegradability in marine waters of textiles made of polylactic acid, a diffused industrially compostable plastic. The test was extended also to cellulose-based and conventional non-biodegradable oil-based plastic textiles. The analyses were complemented by bio-reactor tests for an innovative combined approach. Results show that polylactic acid, a so-called biodegradable plastic, does not degrade in the marine environment for over 428 days. This was also observed for the oil-based polypropylene and polyethylene terephthalate, including their portions in cellulose/oil-based plastic blend textiles. In contrast, natural and regenerated cellulose fibers undergo complete biodegradation within approximately 35 days. Our results indicate that polylactic acid resists marine degradation for at least a year, and suggest that oil-based plastic/cellulose blends are a poor solution to mitigate plastic pollution. The results on polylactic acid further stress that compostability does not imply environmental degradation and that appropriate disposal management is crucial also for compostable plastics. Referring to compostable plastics as biodegradable plastics is misleading as it may convey the perception of a material that degrades in the environment. Conclusively, advances in disposable textiles should consider the environmental impact during their full life cycle, and the existence of environmentally degradable disposal should not represent an alibi for perpetuating destructive throw-away behaviors.

## Introduction

The production and consumption of plastic materials have increased dramatically in the last three decades [1,2]. One of the primary reasons such polymers are employed widely across different applications and represented as the ultimate commodity of convenience is their unique properties, such as their durability, and stability against chemicals, hydrolysis, temperature, light, and microbes [3]. However, these convenient materials are also one of the world’s greatest environmental problems. On the one hand, their essential characteristics are important advantages for synthetic materials. Still, on the other hand, they generate consequent drawbacks when plastic waste enters the environment via inappropriate or careless disposal but also during the everyday weathering of plastic products and materials. Indeed, such unrivaled functional properties result in plastics not degrading and persisting in the natural environment for decades to centuries [4].

The recalcitrance of conventional plastics to environmental degradation represents an immense challenge for our society. The permanence of plastic, especially in its microplastic forms, causes immediate harm and toxicity to the environment and has the potential for long-term, less predictable impacts on global ecosystems and even climate change [5–7]. Microplastics in water bodies, for instance, offer a new, unexplored, floating niche with the potential to spread over long-distance invasive or pathogenic microbial species [8]. Despite the isolating circumpolar frontal systems, floating macro- and micro-plastics are now reported even in the Antarctic region [9]. Such surface plastics are even known to contribute to greenhouse gas emissions as a result of photochemical degradation [7]. Finally, positively buoyant plastics are just the tip of the iceberg, as a large proportion of the waste is estimated to reach deep-sea waters [10], where the fate and ecological impact of macro- and micro-plastics remain primarily unknown [8].

Clothes made out of plastic fibers and nonwovens, such as products for hygiene applications (i.e., wipes, diapers, tampons), are an integral part of the growing plastic production and its subsequent pollution issue [11]. During the 1990s, oil-based plastic started dominating the fiber market and overtook cotton production. In 2020, 59.7 million tons of plastic fibers were produced, making up ca. 62.5 percent of global fiber production [12]. Still, while addressing the general topic of plastic pollution and quantifying its main contributors, very few studies dealt with the impact of the nonwoven and synthetic textiles discarded into the environment, including the fibers released during the use of textiles and the laundering of clothing [13]. Addressing the severe environmental threat offered by synthetic materials, researchers made significant progress in elaborating alternatives and developing biodegradable materials [14,15].

Polylactic acid (PLA) is a leading polymer in the plastics market. It is mainly produced from renewable natural resources and is regarded as a representative of the so-called biodegradable plastics [5,16,17]. PLA is considered an alternative with similar functionalities to that of conventional oil-based plastics, which on the other hand, tend to be non-biodegradable. However, invoking biodegradability in the context of plastic disposal and pollution requires particular care [18,19]. By definition, biodegradability is the property of a material to undergo degradation down to water, carbon dioxide, methane, basic elements, and biomass, by the action of living organisms [20]. The biodegradation of plastic materials within a desirable time frame requires specific conditions of light, humidity, temperature, and availability in oxygen, nitrogen, and microbial communities, [18,19,21–24] that are achieved in industrial composting settings, but are not representative of the natural environments [5]. Moreover, nature comprises a much wider complexity and diversity of interplaying physical and biological factors that change in both space and time, so for a material to be defined as universally biodegradable in the environment is impossible. Biodegradability in the environment is, thus, better defined as a system property, which takes into account the interaction between the attributes of the specific material with those of the specific receiving environment. In other words, each environment would have its peculiar biodegradability potential for each type of plastic.

Because of the existing mismatch between conditions achieved in controlled settings and those occurring in natural environments, the material’s biodegradability assessed in the first scenario might not imply similar performances in the second. The performance approval of products being tested for marine biodegradability in controlled settings, might not be representative and realistic in the ecological sense [18]. This raises doubts about the potential of so-called biodegradable plastics, tested in controlled settings, to end up as environmental contaminants [17,25].

To test environmental degradability, several studies have looked at the fate of so-called biodegradable plastics in experimental settings representative of marine sediments [26], composting plants [21,27,28], cold marine habitats [29,30], and soil burial conditions [28]. In the last years, a growing number of works investigated plastic degradation directly in the marine environment [15,17,31–36], with results underlining a significant variability in their degradation performances [37,38]. Recently, further works investigated the aerobic biodegradation of different plastic polymers [39–41]. Still, most of these tests rely on reaction vessels (or bioreactors), representing a closed system very different from any natural conditions. Although results may be similar in the environment, this type of study is incomplete and cannot be extrapolated to natural marine conditions [18,42]. Overall the current knowledge on the biodegradation of plastic materials in marine and freshwater environments is still limited, and experimental data vary strongly, reflecting the diversity in the employed testing techniques and the duration of the tests [43]. So, while tests performed directly in the marine environment are increasing, comparing their results with laboratory-based ones remains difficult. Our work represents one of the few pioneer studies [43,44] addressing this comparability and stressing the need for reliable, and as much as possible standardized, environmental tests to be developed in parallel to validate laboratory-based measurements. Only in this way will it be possible to evaluate these materials’ environmental risks vs. benefits realistically, allowing an assessment of their complete life cycle.

In the present study, we used the combination of field and laboratory observations to test the environmental degradation of bio-based PLA in comparison to some non-biodegradable oil-based conventional plastics typically employed in the textiles and nonwoven industry. Bioreactors tests were conducted to assess the biodegradability for 28 days at 30°C in closed systems where carbon dioxide was captured while *in situ* surface and seafloor seawater experiments were conducted in Californian coastal waters to assess environmental degradation and complete disintegration based on weekly sampling. PLA was chosen given its extensive application in single-use plastic items and textile material as a replacement for conventional oil-based plastics [45,46]. Polyester and polypropylene (PP) were proxies for conventional oil-based polymers, given their widespread use for synthetic materials. More precisely, polyethylene terephthalate (PET) was the type of polyester used for this experiment. Natural and regenerated cellulose fibers including organic (OCO) and non-organic virgin cotton (NOCO), Lyocell (CLY), Modal (CMD), and Viscose (CV), as well as 50–50 blends CLY/PET and CLY/PP were also tested.

Collected data for these selected materials indicate that natural and regenerated cellulose-based fibers completely degrade in approximately 35 days, while PLA-based, PET, and PP, including the one in blends, did not show any visible sign of biodegradation for the entire duration of the experiments (428 days). Careful inspection of Raman spectra indicated no chemical changes for PLA, PET, and PP (including their portion in the blends) while scanning electron microscopy (SEM) demonstrated the lack of biofilm growth on these materials. Altogether, we conclude that increasing the use of PLA and 50% oil-based blends might not be a realistic alternative to reduce plastic pollution due to the poor assimilation of these polymers in marine environments. In contrast, cellulose-based materials offer the benefit of a short residence time in the marine environment where the materials are being used as a source of carbon and nutrition for the microbial communities. This benefit, however, must be evaluated in the context of sustainability and potential pollution at all the phases of the cellulose fibers production cycle [47], and should not offer an excuse for the perpetuation of throw-away behaviors of these particular materials.

## Material and methods

Study site. Material samples tested for disintegration were submerged at the sea surface and seafloor levels (10 m depth) along the Ellen Browning Scripps Memorial Pier (ca. pillar 31) located at Scripps Institution of Oceanography (SIO) in La Jolla, California (Lat: 32.866501 N, Long: 117.254583 W; S1 Fig).

Experimental design. The sea surface experiment consisted of four metallic cages used to contain the material samples that were kept in individual double mesh Nylon pockets of 710 μm. For each sample type, a rectangle (9 cm x 5 cm) and a square (5 cm x 5 cm) were used to follow the disintegration and the integrity of the fibers, respectively (S2A Fig). The rectangle was placed in an inner Nylon pocket, while the square was placed in a similar but larger Nylon pocket that contained a tag identifier with a number engraved in a stainless-steel coin and the matching rectangle sample (S2B Fig). The seafloor experimental design consisted of submerging the material samples at 10 m depth on the sediment surface, at the sediment-water interface, where each sample had its designated screw-on polyvinyl chloride (PVC) cage reinforced with 316 stainless steel material (S3A Fig).

For each experiment, the samples were removed from the experimental sites every seven days, gently rinsed using ambient seawater, and kept wet until further treatment (imaging and/or subsampling). Images of the rectangle sample were taken while a portion of the square sample was cut and kept aside for scanning electron microscopy (SEM) analysis. After the analysis, lasting no longer than three hours, the samples were put in random order into their compartment and back in the seawater. For each material sample completely disintegrated, a replacement of the same material was used for the replicability of the data and at least tested a minimum of three times consecutively over the whole duration of the experiment. The sea surface experiment began on March 29, 2019 (D0), and ended on November 15, 2019 (D231), while the seafloor experiment started on August 16, 2019 (D0) and ended on March 5, 2020 (D202). After resuming the field experiment, the samples that were not completely disintegrated were relocated to the experimental aquarium facility on the SIO campus for another 197 days. The aquarium was equipped with water tables with open-circuit seawater circulating directly from the field. The seawater was collected about three meters below the surface at the end of the SIO pier, and filtered through a large sand filter by percolation before being distributed to buildings across campus. The seawater reflects coastal waters’ properties but contains fewer macro particulates (mainly less zoo- and phytoplankton).

Sample selection. The different types of fabrics used for all experiments were regenerated and natural cellulose, bio-based plastic, oil-based plastic, and blend materials described in Table 1. The morphology of the nonwoven fabrics was comparable using a consistent ~ 300–600 needle punches per cm^2^ and a basis weight between 55 and 70 gsm. The sources for each type of material are listed in Table 1.

**Table 1:** Description of the different materials used for the sea surface, seafloor, and biodegradation experiments including the category, name, acronym, titer (dtex), length of the fiber (mm), needle punches (/cm^2^), weight (g/m^2^) and the provider.

Categories	Name	Acronym	Titer (dtex)	Cut length of fibers used (mm)	Needle punches (/cm^2)	Basic weight of nonwoven (g/m^2)	Source
Regenerated cellulose	Lyocell	CLY	1.7	38	600	60	Lenzing
	Modal	CMD	1.3	39	600	60	Lenzing
	Viscose	CV	1.7	40	600	60	Lenzing
Natural cellulose	Organic Virgin Cotton	OCO	2.0	25	400	80	Tosama
	Non-organic virgin cotton	NOCO	2.0	25	300	60	Tosama
Blend	50% Lyocell 50% Polyethylene terephthalate	CLY/PET	CLY: 1.7 PET: 1.7	CLY: 38 PET: 38	600	55	Lenzing/Trevira
	50% Lyocell 50% Polypropylene	CLY/PP	CLY: 1.7 PP: 1.7	CLY: 38 PP: 38	600	55	Lenzing/Fiber Visions
Bio-based plastic	Polylactic acid	PLA	1.3	38	600	55	HXN
Oil-based plastic	Polyethylene terephthalate	PET	1.7	38	600	55	Trevira
	Polypropylene	PP	1.7	40	300	55	Fiber Visions

NA refers to Non-Applicable.

Raman spectroscopy analysis. Raman spectroscopy on individual untreated microfibers was performed at room temperature with an In Via Renishaw Confocal Laser Raman system equipped with a 514.5 nm laser source. Punctual data with a spot size of about 1–2 μm, were collected using 50X, 100X objectives (N.A. = 0.75; 0.85) on a Leica DM LM microscope and a monitored laser power at the sample surface of 0.5–5 mW for 20–120 s of total acquisition time. New spectra were recorded in the case of high fluorescence background or laser-induced photothermal damage. This ensured good replicability and quality of the measurements for every sample. The investigation on degrading materials was performed through the comparison of their spectra against the within-sample spectral heterogeneity measured at D0 for a total of 501 spectra evaluated for this study. Bio-based plastic (PLA), blend (CLY/PET and CLY/PP), and oil-based materials (PET and PP) were analyzed at D84, while cellulose-based materials were analyzed at D7 (CMD and CV), D14 (CMD and CV), D21 (CLY and OCO), D28 (CLY, OCO, and NOCO) and D35 (NOCO).

Spectra on bio-based plastic and oil-based plastic fibers have been processed for curve fitting using Crystal Sleuth [48] and OriginPro 8.5. Obtained parameters and calculated ratios, representative of the polymer’s degree of crystallinity and polymerization, have been used to test the hypothesis of structural degradation through paired t-tests (D0 vs D84) on groups size of 10 pairs (α = 0.05). Further methodological details can be found in the Supplementary Information. In addition, the spectra obtained at D0 allowed definitive identification of the individual bio-based plastic and oil-based materials (PLA, PET, PP), while cellulose-based fibers were distinguished only in relation to their crystal lattice types, cellulose II for regenerated cellulose fibers (CLY, CMD, and CV) and cellulose I for natural cellulose fibers (OCO and NOCO), with no further discrimination between the original production processes (S4 Fig and bands assignments are shown in S1 Table).

SEM imaging and fiber diameter analysis. Samples of fabrics freshly collected from the field were dried in an open Petri dish inside a protected area (fume hood). A small piece (approx. 5x5 mm) of the sample was cut off using a razor blade and placed on double-sided carbon tape on SEM Stubs (EMS®). The small samples were then exposed to regular conventional SEM processing for viewing. The samples were first coated with gold-palladium in a sputter coater following the manufacturer protocol (EMS®) before being observed using a Zeiss EVO10 SEM. Images captured at 165/175x, and 485/495x were used to measure the diameter of fibers. Measurements were made in ImageJ from at least 10 images (5 per magnification) in which only fibers in focus were measured in diameter. Only one measurement was performed per fiber, that is until it was not possible anymore to follow the fiber along its length (because of going out of focus or getting covered by other fibers). When biofilm was present, diameter measurement was made from part of the fiber where the biofilm was the thinnest (n = 60 for each sample). The difference in the fiber diameter was tested for significance using an unpaired t-test after data distribution was tested for normality and equal variances using the Shapiro-Wilk test and Bartlett’s test.

Bioreactor experiment. ASTM D6691 [49], a standard test method used for determining the aerobic biodegradation of plastic materials in the marine environment was used to assess the biodegradation of the sea surface and the seafloor samples. The tests were performed by Organic Waste Systems (OWS®) in Belgium, and the total test duration was 28 days. The experiment was performed in duplicate and the incubation temperature was 30°C ± 2°C. The control reactors contained 250 g of enriched seawater where 60 mg of commercial powder of microcrystalline cellulose was added and used as the reference material (Sigma Aldrich, Avicel® PH-101; particle size ~ 50 μm). The used inoculum was derived from natural seawater collected from the open sea in Belgium. The natural seawater contained an indigenous population of microorganisms and was enriched with inorganic nutrients (0.05 g/L NH_4_Cl and 0.1 g/L KH_2_PO_4_). The medium was pre-incubated for four days at 30°C ± 2°C and sieved on an 80 μm screen. After adding the reference and test items (the loose fibrous form obtained from chopping), the reactors were put on an inductive stirrer. A magnetic rod kept the reference item, the test items, and the growing biomass in suspension during the test.

Briefly, the % of CO_2_ produced by a defined microbial consortium is used here as a proxy for the evolution of the average biodegradation in % of reference and test materials.

During the aerobic biodegradation of organic matter in a marine medium, O_2_ is consumed, and polymer organic carbon is converted into gaseous CO_2_. However, part of the carbon is not released as CO_2_ but is used for cell growth. The produced CO_2_ is trapped by KOH solution, and the induced pressure drop is directly related to the consumed O_2_ and hence to the biodegradation of the test material. The biodegradation based on CO_2_ production is calculated as the percentage of solid organic carbon of the test item which is converted to gaseous CO_2_. A value of 100% absolute biodegradation cannot be achieved due to a small fraction of carbon consumed for the cell growth of microorganisms, which prevents its release as CO_2_. The test is considered valid if the reference cellulose is more than 70% biodegraded at the end of the experiment (ASTM D6691).

Environmental parameters. Parameters collected at the SIO pier comprised wind speed (m/s), wave height (m), and surface seawater temperature (°C). The high-resolution data (every 0.5 s) spanned from January 2019 to May 2020 and were averaged daily. The time series are presented in S3 Fig. The data were provided by the Shore Stations Program sponsored at SIO by California State Parks, Division of Boating and Waterways, and also collected by the Birch Aquarium at Scripps staff and volunteers.

Statistical analysis. Matlab R2015b and the Python libraries Matplotlib and Seaborn were used for the statistical analysis and figure construction. Student-test was conducted to detect significant differences between the material categories and samples with a significance level α = 0.05. Pair comparisons between the material types were performed using a non-parametric model N-way analysis of variance was used to examine the influence of categorical independent variables material type (mt), disintegration time (dt), and experiment type (et) on one continuous dependent variable (wind speed (WS), wave height (WH) or seawater temperature (SST)). The ANOVA analysis helped to assess the main effect of each independent variable and test the presence of any interaction between them. The anovan function in Matlab was used (e.g. anovan(SST,{mt dt et})). Differences were considered significant when the calculated p-value was below 0.05. For each dependent variable, a multivariate linear model was also computed using dt, and WS, WH or SST as discrete variables (e.g. fitlm(SST, [mt, dt, et], ‘CategoricalVars’, {mt, et}). Additional analyses were also conducted to assess whether or not there were significant differences between the diameters of independent fibers between different time points using Man-Whitney one-sided, with a significance level α = 0.05.

## Results

### Coastal conditions facilitate the rapid fragmentation of fabrics made only of cellulose-based materials

To estimate the average time necessary for fabrics to disintegrate under natural conditions, we obtained nonwoven materials of different origins (bio- and oil-based plastics, cellulosic fabrics, and blends) and exposed them to the coastal sea surface and shallow seafloor (10 m depth) conditions.

**Fig 1:**
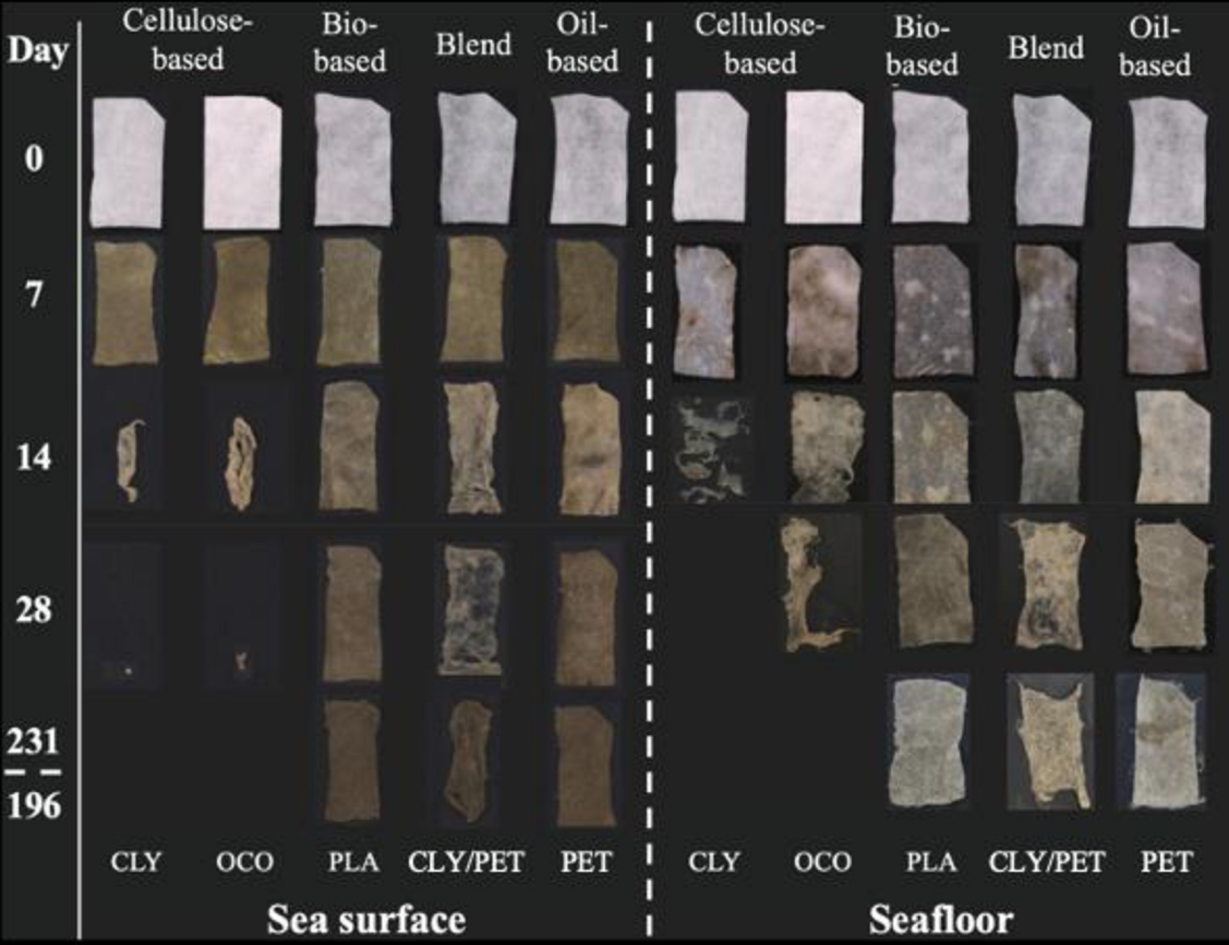
Disintegration time in days for five selected types of material exposed to coastal waters at the Ellen Browning Scripps Memorial Pier located at Scripps Institution of Oceanography in La Jolla, California. The degradation time corresponds to the last measurement day showing no material within the experimental compartment. Samples were kept for a total of 231 days at the sea surface and 196 days at the seafloor. The different materials exposed fall under the following categories: *Cellulose-based* with regenerated cellulose material represented by Lyocell (CLY) and natural cellulose material represented by organic virgin cotton (OCO), *bio-based plastic* material represented by polylactic acid (PLA), conventional *oil-based* material represented by polyethylene terephthalate (PET) and *blend* material represented by 50% CLY and 50% PET. The samples for the sea surface experiment were submerged within the first 30 cm of the water column while the seafloor samples were deposited at the seafloor bed at ca.10 m depth.

Exemplary and representative images of the materials monitored weekly for the sea surface, and seafloor experiments are shown in Fig 1. The number of days the samples were submerged in natural seawater at the pier of SIO was 231 and 196 days for the sea surface and the seafloor experiments, respectively. All samples showed visual signs of weathering and morphological changes due to their exposure to environmental conditions (Fig 1). Samples showed an alteration in color during the first seven days with a prominent change from white to gray and green for the sea surface experiment. This was attributed to the aggregation of mineral particles (sand) and biofilm formation. In the seafloor experiment, we observed a more subtle change from white to gray tones related to the presence of mineral grains (Fig 1).

Cellulose-based samples were fragmented on average within a month (30±11 days), with a mean of 30 days (±9 days) for the sea surface and 30 days (±14 days) for the seafloor experiments (Table 2). This fragmentation time for the sea surface experiment ranged from 24±4 days and 40±8 days for CV and OCO, respectively, while for the seafloor experiment, the results ranged from 22±8 days and 41±7 days for CV and NOCO, respectively (Table 2). There was no significant difference (t-test, p-value > 0.05) observed between the disintegration time within the regenerated cellulose fabrics (CLY, CMD, and CV) and no significant difference (t-test, p-value > 0.05) within the natural cellulose fabrics (OCO and NOCO) either. However, a significant difference (t-test, p = 0.0429) was observed for the sea surface experiment between the regenerated (n = 13) and natural (n = 8) cellulose materials averaged together with a slower disintegration time for OCO and NOCO (mean of 34±11 days) compared to CLY, CMD, and CV with a mean of 26 days (±7 days). Similarly, the seafloor experiment showed a significant difference (t-test, p = 0.0085) between the regenerated (n = 15) and natural (n = 9) cellulose materials averaged together with a consistently slower disintegration time for OCO and NOCO (mean of 40 ±16 days) compared to CLY, CMD, and CV (mean of 24 ±9 days; Table 2).

**Table 2:** Disintegration time in days for the sea surface and the seafloor experiments and % of biodegradation achieved after 28 days of incubations for the bio-reactor experiments. The degradation time corresponds to the day of the last measurement showing no material within the experimental compartment while the % of biodegradation is based on the % of CO_2_ produced after 28 days of incubations.

Sample materials	Categories	Sea surface		Seafloor		Bio-reactor	
		Avg (days)	Std (days)	Avg (days)	Std (days)	Avg (% biodegradation after 28 days)	Std (% biodegradation after 28 days)
Lyocell (CLY)	Regenerated cellulose	26.3	8.8	25.0	10.6	76.0	2.7
Modal (CMD)		29.8	6.7	25.0	10.6	81.4	2.0
Viscose (CV)		23.8	3.8	22.2	7.5	82.2	1.2
Organic Virgin Cotton (OCO)	Natural cellulose	30.8	11.7	41.7	7.0	81.2	0.0
Non-Organic Virgin Cotton (NOCO)		39.7	8.1	29.8	10.3	NA	NA
50/50 Lyocell/Polyethylene terephthalate (CLY/PET)	Blend	ND	ND	ND	ND	NA	NA
50/50 Lyocell/Polypropylene (CLY/PP)		ND	ND	ND	ND	NA	NA
Polylactic acid (PLA)	Bio-based plastic	ND	ND	ND	ND	-0.2	2.0
Polyethylene terephthalate (PET)	Oil-based plastic	ND	ND	ND	ND	-0.3	2.0
Polypropylene (PP)		ND	ND	ND	ND	-0.8	0.3

ND refers to material samples that did not disintegrate (i.e. Non-Disintegrated).

NA refers to Non-Applicable.

On the other hand, none of the bio-based (PLA), oil-based (PET and PP), and blend (CLY/PET, and CLY/PP) showed any sign of disintegration (or onset of breakdown) for the entirety of the experiments (Fig 1 and Table 2). This corresponds to more than 231 and 196 days exposed to environmental conditions for the sea surface and seafloor experiments, respectively. Visible signs of fragmentation and/or fabric thinning were sometimes observed, but only for blend samples (CLY/PET and CLY/PP) (Fig 1). Although showing translucent areas due to progressive thinning of some of the material with heterogeneous thickness, the blend materials remained in their original shape until the end of both experiments. Similar data were observed for PLA, PET, PP, and the blend materials that were kept in open-circuit aquariums, beyond the 231 days in the field, with the same natural seawater flowing, for up to 428 days where they looked very similar to the material exposed to complete natural conditions.

**Fig 2:**
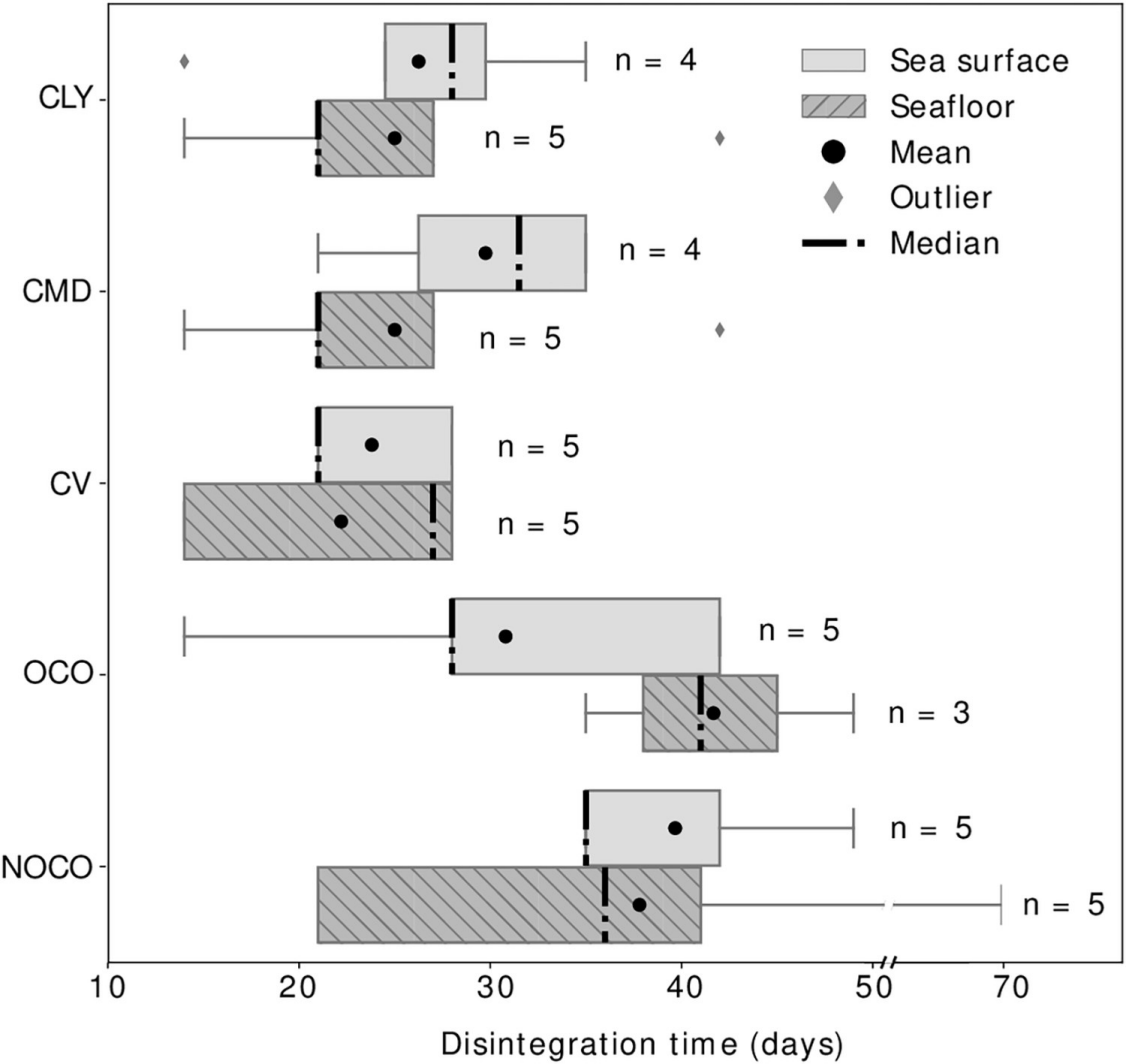
Comparison of the disintegration time in days between the samples submerged at the sea surface (light grey) and the seafloor levels (dark grey with dashed lines). The spacings between the different parts of the box indicate the degree of dispersion and the skewness in the data. Please note that the regenerated cellulose materials Lyocell (CLY), Modal (CMD), and Viscose (CV) and the natural cellulose materials organic virgin cotton (OCO) and non-organic virgin cotton (NOCO)) are the materials that showed a complete disintegration and hence are the only materials represented here. The bolded dashed line represents the median while the black dots represent the mean. Datapoints that fall outside of the upper and lower quartiles and the whiskers are represented by a diamond shape symbol.

Fig 2 is the visual representation of the variability of the degradation time within each material type for the sea surface and the seafloor experiments, while the total variability of each experiment is shown in S5 Fig. For most material types, the mean and median fall very closely, showing a symmetrical distribution of the data.

### Chemical weathering was detected using Raman for cellulose-based fabrics

Raman spectra were collected on weathered samples and compared to virgin materials at Day 0 (D0) to investigate the structural-chemical degradation of the fibers. Samples composed of cellulose-based fabrics have been measured at the last two sampling steps of their complete biodegradation cycle (D21 and D28). PLA, conventional oil-based plastic (PET and PP) and blend (CLY/PET and CLY/PP) materials have been measured at D84 only. Results on weathered samples evidenced significant changes in the spectra of cellulose-based materials during the studied time frame while bio- and oil-based plastic fibers remained unchanged.

**Fig 3:**
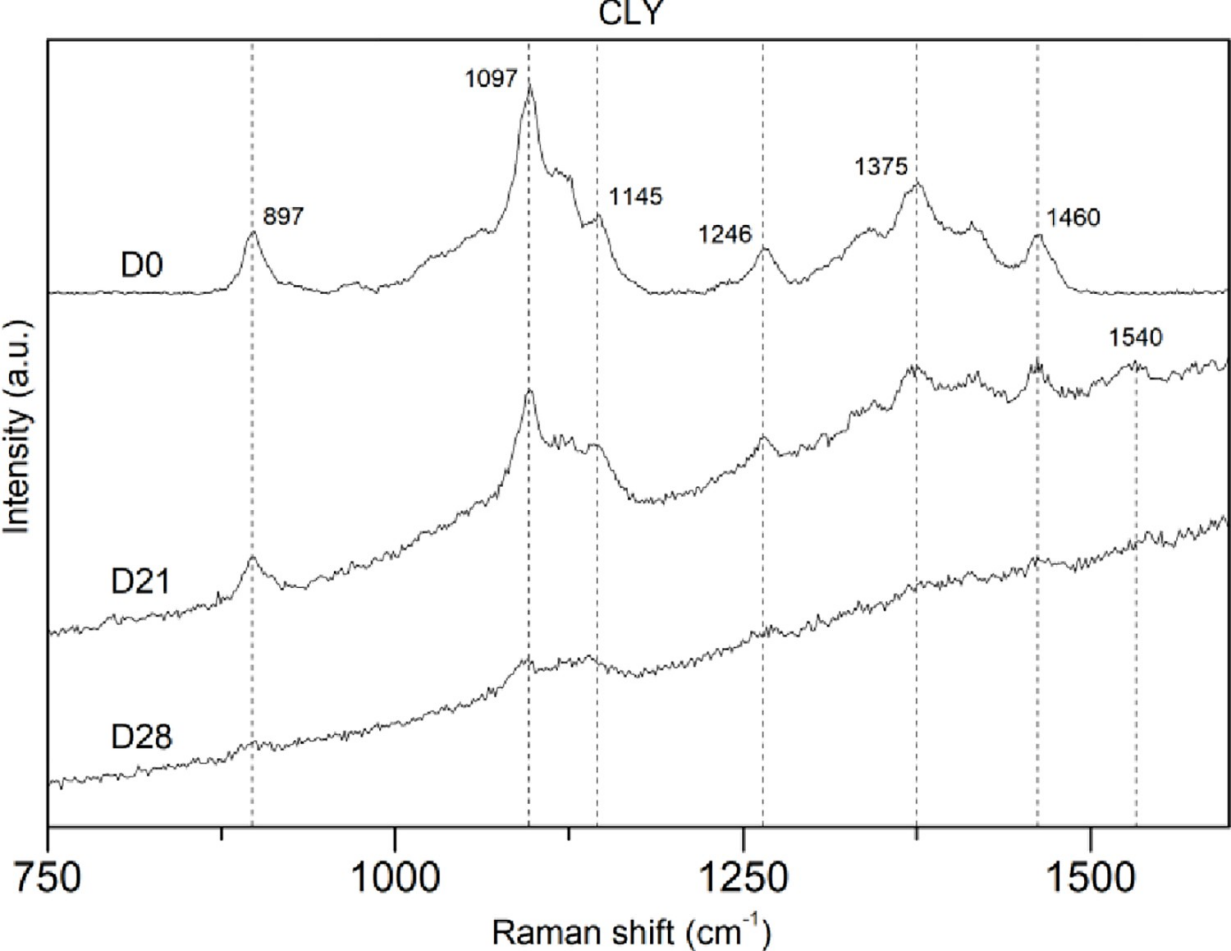
Variability in spectra using Raman Spectroscopy for replicates of Lyocell (CLY) samples at several time intervals. **D0.** Visually and morphologically intact fiber not exposed yet to environmental and oceanic conditions; **D21.** Visually and morphologically well-preserved fiber after 21 days of exposure to oceanic conditions; **D28.** Well-altered fiber was observed after 28 days of exposure to coastal waters. Please note that the selected spectra had a similar fluorescence background.

On the one hand, spectra on cellulose-based fabrics (CLY, CMD, CV, OCO, and NOCO) showed a general trend characterized by a decreased intensity at all peaks coupled to a fluorescence background gradually increasing with wavenumbers (Fig 3). This suggests structural loss and growing “molecular impurities”, including the increase of chromophores formation [50]. Such a decline of the cellulose structural signal correlates with an increasing morphological alteration, including yellowing and “embrittlement” at the analyzed individual fibers. Furthermore, co-existing with this general progressive alteration, cellulosic materials showed, at each analyzed step, some degree of within-sample heterogeneity (i.e., differences between the individual fibers and, on some occasions, within the same fiber) in both visual and structural preservation. This was proved by the occurrence of well-preserved fibers, resulting in D0-like spectra, even at the last sampling steps, just before the completion of the degradation cycle.

**Fig 4:**
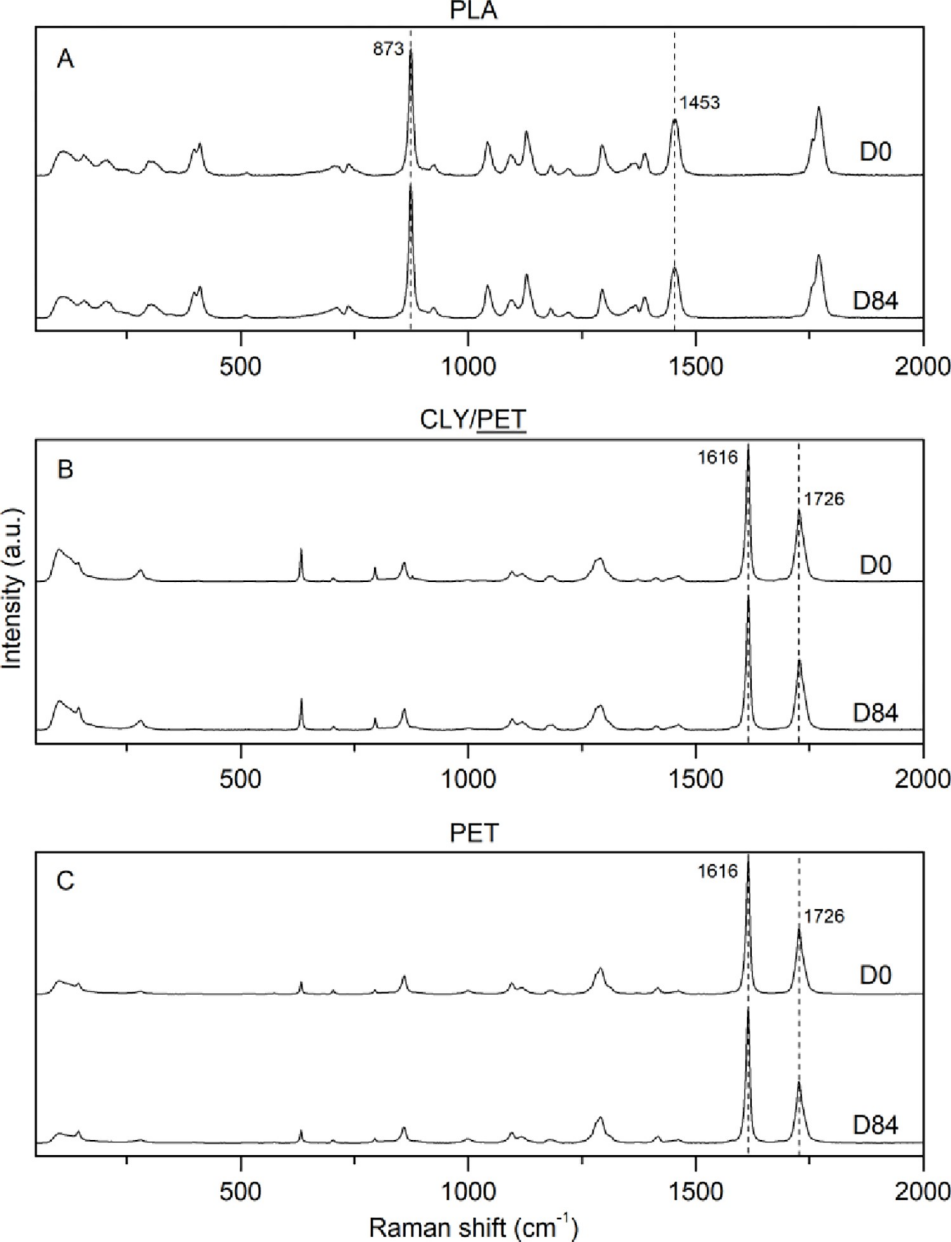
Raman spectra at Day 0 and Day 84 for A. Polylactic acid (PLA); B. Blend sample comprise of Lyocell (CLY) and polyethylene terephthalate (PET), where only PET is represented and was the only representant remaining at Day 84; C. PET. Note that variations in the peak’s relative intensities between B and C are related to different fibers orientation under the laser and thus do not account for any structural change.

On the other hand, spectra on oil-based and blend materials at D0 and D84 were evaluated qualitatively but also semi-quantitatively through parameters and ratios obtained after curve fitting. Except for changes in relative intensities resulting from anisotropic effects under Raman excitation, falling within the range of the D0 within-sample spectral variability, the profiles of PLA, PET, and PP at D84 were a perfect replica of those recorded at D0. The spectra showed unvaried peaks occurrences, positions, and shapes (Figs 4A, 4C and S6). The same results were observed for blend materials, whereas after 84 days well-preserved oil-based fibers were solely detected, while no cellulose-based (CLY) remains were found, which was the case for more than 100 different spectra (Figs 4 and S6). An extra step was also taken where the blend fabric samples were ripped apart to create a powder of individual fibers before Raman analysis. Yet, only conventional oil-based and PLA fibers were detected (i.e., all cellulose-based material was then wholly gone).

Calculated parameters and ratios reflecting the degree of polymerization and crystallinity of PLA, PET, and PP showed no significant variation (t-test, p-value > 0.05) for their mean values between D0 and D84 (S1 Table); including PET and PP in the blend samples. This confirmed the preservation of the original oil-based fibers’ chemical integrity concerning the structural arrangement of polymer crystals.

### The structural integrity of the bio-based, oil-based, and blend fabrics was related to the lack of a biofilm

**Fig 5:**
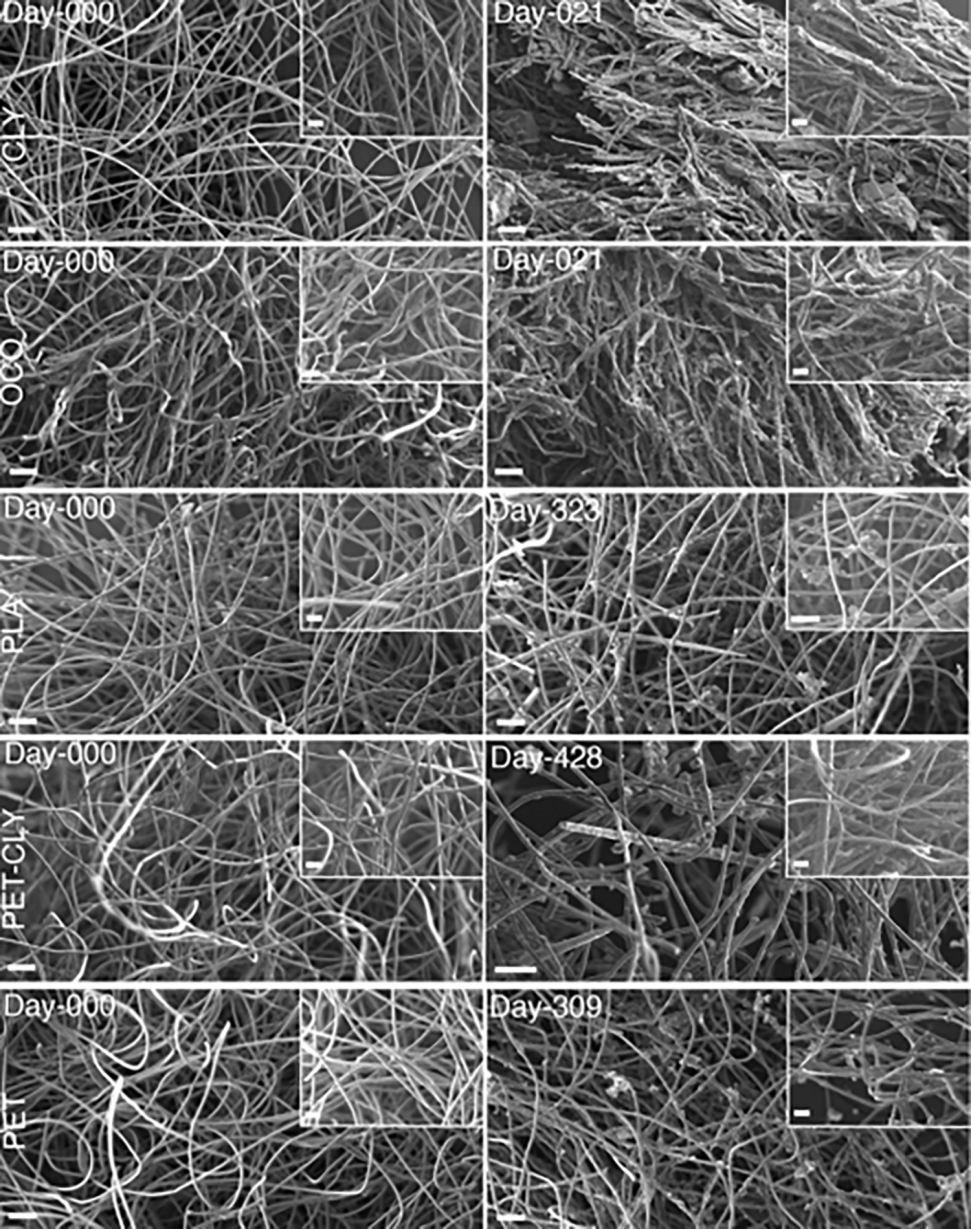
Scanning electron microscopy (SEM) images for the following sample categories before (Day 0) and after (Day X) exposure to sea surface marine conditions: *Regenerated* cellulose material represented by Lyocell (CLY) and *natural* cellulose material represented by organic virgin cotton (OCO), *bio-based plastic* material represented by polylactic acid (PLA), *blend* material represented by 50% CLY and 50% polyethylene terephthalate (PET) and conventional *oil-based* material represented by PET. The samples selected for each category correspond to the samples exhibiting results close to the average disintegration time for the *cellulosic* materials and were randomly selected for the other categories since none of these materials were disintegrated. Main images were all taken at a magnification between 165-175x (scale bar is 100 μm for all panels) while inserts were all taken at a magnification between 485-495x (scale bar is 50 μm for all inserts).

Using SEM we showed that PLA, and conventional oil-based materials remained deprived of any visible biofilm, displaying limited organic material at the surface of their constitutive fibers. Consequently, these fibers appeared very smooth and clean, showing no sign of weakening of structural integrity (Fig 5). In contrast, cellulose-based material showed signs of biodegradation by displaying dense organic material embedded with the fibers (Fig 5). This biofilm permeated the entire thickness of the fabrics, in which the fibers then showed a significant decrease in the diameter size between D0 and D21 for both CLY (Man-Whitney one-sided, p = 0.00014) and OCO (Man-Whitney one-sided, p = 0.01) with a corresponding decrease in fiber diameter of 14% and 8% observed for CLY and OCO, respectively. As for the bio- and oil-based fibers, no significant decrease in diameter was observed (Man-Whitney one-sided test; p-value > 0.05). An exception comes from the blend materials that showed, in some instances, fibers completely covered with biofilm deep inside the fabric.

### Biodegradation of cellulose-based fabrics demonstrated in a close system

**Fig 6:**
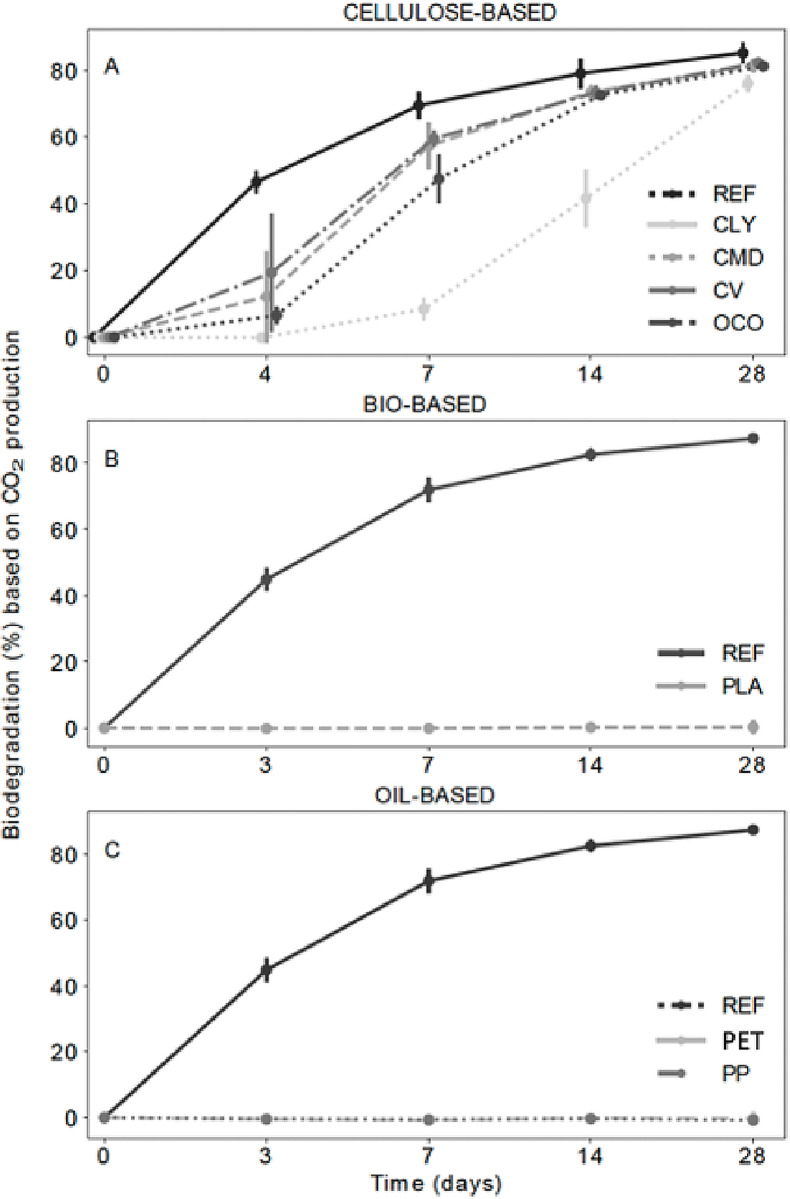
Evolution of the average biodegradation in % based on CO_2_ production using the ASTM D6691 standard test method for the following material types: A. Cellulose reference, Lyocell (CLY), Modal (CMD), Viscose (CV), and organic virgin cotton (OCO). B. Cellulose reference and polylactic acid (PLA). C. Cellulose reference, polyethylene terephthalate (PET), and polypropylene (PP). The error bars represent the variability within the experiment for duplicate samples.

To ensure that the disappearance of the different material samples exposed to marine conditions was not the sole result of the mechanical degradation of the material over time, biodegradation experiments were carried out in closed systems by OWS®, a third-party running environmental test laboratory. These consisted of using bio-reactors where biodegradation was determined by measuring the amount of CO_2_ produced and captured in a potassium hydroxide (KOH) solution during the test. Results show that after 28 days of incubation, biodegradation of 85.2% ±3.8% was measured for the cellulose reference material (S2 Table). The biodegradation curves of the materials are shown in Fig 6. Cellulose-based samples show a similar biodegradation trend as the cellulose reference material. This corresponds to absolute biodegradation >76.0% relative to the reference material after 28 days (Fig 6A). The biodegradation rates of CMD, CV, and OCO were slower during the first four days of the experiment, increased from D4 to D7 and then slowed down until Day 28 (Fig 6A, S2 Table). This profile was slightly different for CLY, which showed no biodegradation during the first four days, after which biodegradation proceeded at a fast rate (Fig 6A, S2 Table). Overall, although not statistically different from each other’s (t-test, p-value > 0.05) the absolute highest biodegradation level after 28 days was attributed to CV with 82.2% (±3.8%), followed by CMD with 81.4% (±2.0%), OCO with 81.2% (±0.0%) and CLY with 76.0% (±2.7%).

In contrast, no significant biodegradation (t-test, p-value > 0.05) was observed after 28 days for the material samples PLA, PET, and PP throughout the test reflected in an absolute value of 0.2% (± 0.2%), -0.3% (± 2.0%) and -0.8% (± 0.3%), respectively (Fig 6B and 6C, S2 Table). The negative results observed for the material samples PET and PP are because of the low CO_2_ production in the test reactor as opposed to the control reactors causing a negative net signal and hence a negative result.

### For cellulose-based materials, fragmentation in the field was not significantly affected by local environmental conditions

Environmental conditions were variable across 2019 and 2020 where daily averaged wind speed ranged from ca. 0.6 m/s to 6.9 m/s and daily averaged wave height ranged from 0.3 m to ca. 2.0 m high (S7A Fig). Sea surface temperature was also variable across seasons with an average temperature of 17.5°C overall and 14.2°C during the winter months (December to March) and an average temperature of 22.3°C during the summer months (S7B Fig). None of the variations in these parameters could be correlated with the variability of fragmentation time observed across samples, conditions and along the timeline of the experiment.

## Discussion

A combination of *in situ* coastal experiments along the California Coast at the sea surface and seafloor (14–24°C), bio-reactor experiments (28 days at 30°C), Raman microscopy, bright field, and SEM imaging revealed that cellulose-based fibers biodegrade in less than one month. At the same time, the bio-based plastic PLA, and the oil-based PET and PP, including their component in blend textiles, show a longer endurance and may reside in the marine environment for an undetermined amount of time, representing an important source of anthropogenic pollution. Particularly, bio-based PLA, an industrially compostable polymer, so-called biodegradable plastic, showed no sign of environmental degradation after 428 days under natural marine conditions. Such results emphasize the existing discrepancy between compostability and environmental degradability, stressing the potential of compostable plastics as marine pollutants.

### Providing environmentally biodegradable alternatives to plastics requires testing across many different conditions and environments

Various types of so-called biodegradable plastics have been proposed as a solution to lessen the environmental impacts of plastic debris and are being introduced into the market with a growing share in recent years [22,23,51]. These materials are typically certified for biodegradation in industrial composting, in the presence of ideal conditions for the breaking of chemical bonds (i.e., high temperatures). However, the fate of such compostable plastics when entering the natural environment remains poorly explored. Indeed, the proof of environmental degradation beyond controlled laboratory test conditions should be the first prerequisite for a material to be considered a valuable alternative to non-biodegradable materials in a specific natural environment but is not yet a common practice. While standard tests (ASTM, ISO, EN) are available for industrial and home compostability, and also in closed systems for fresh and marine waters [52,53], few methods have been developed to assess degradation in real marine environments (ISO 22766:2020 Plastics—Determination of the degree of disintegration of plastic materials in marine habitats under real field conditions and ISO/AWI 16636 Plastics—Simple field test of disintegration of plastics under real marine environment). It is also well recognized that positive experimental results on the biodegradation of materials in one environment (i.e. in industrial compost) do not automatically imply comparable or sufficient biodegradation rates of that material in another system (i.e. in the marine environment) [18,51,54,55] and hence the concept of biodegradability needs to be seen as an ecosystem capacity considered in synergy with the inherent bulk properties of the plastics. These results must be discriminated against accordingly and be case-specific, and the information must pass on to the consumer as well.

Since biodegradation results measured in the lab in closed systems indicate potential but are not absolutes in nature, conducting experiments under natural conditions in the field to support data generated using bio-reactors should become a prerequisite. In our study, we observed similar degradation times for the *in situ* experiments conducted in the coastal waters of Southern California (disintegration) and the bio-reactor experiments (bio-degradation—ASTM D6691). Yet, given a specific set of conditions and environmental scenarios, there is not a clear biodegradation threshold to qualify a material as biodegradable in marine environments [40,56] except for that established by ISO 22403:2020 Plastics—Assessment of the intrinsic biodegradability of materials exposed to marine inocula under mesophilic aerobic laboratory conditions—Test methods and requirements https://www.iso.org/standard/73121.html. However, our parallel experiments provided a similar time frame for the biodegradation and degradation of cellulose-based material in less than a month compared to the bio-based, conventional oil-based, and blend materials that remained intact. In general, the profiles were the same in the two experiments: REF > regenerated cellulose (CLY, CMD, CV) > natural cellulose (OCO, NOCO) > (Blend) PLA, PET, PP with significant differences (t-test, p-value < 0.05) between the regenerated (faster biodegradation) vs. the natural (slower biodegradation) cellulose materials.

### PLA, PET, and PP showed no signs of degradation after >400 days in seawater

The differences in the evolution of the Raman spectra between the cellulose-based material, the PLA, and the oil-based materials were remarkable. Particularly, for PLA, Raman spectra of samples weathered for 84 days were a perfect replica of those recorded at D0, showing unvaried peaks occurrences, positions, relative intensities, and shapes. Raman spectra of PET and PP weathered for 84 days also proved identical to virgin materials, highlighting the lack of structural degradation for these polymers. These results were also supported by SEM images, whereas no differences in fiber diameter were observed during the entire course of the experiment. On the other hand, spectra on both regenerated (CLY) and natural (OCO) cellulosic materials showed a general trend characterized by an intensity decrease at all peaks coupled with an increased fluorescence background suggesting structural loss and growing “impurities”. Indeed, the appearance of a new band arising consistently across the cellulose-based weathered materials at 1520–1540 cm^-1^ is likely due to the formation of new molecular structures such as carotenoid-like molecules occurring during cellulose degradation [50,57–59]. In parallel, SEM images for CLY and OCO also reflected the degradation of the cellulose through the decrease in fiber diameter, similar to the specific surface area decrease of natural polymer material occurring during laundering and textile use [60].

Thanks to their lower degree of crystallinity, regenerated cellulose fibers (CLY, CMD, and CV) biodegrade faster than natural cellulose (i.e., cotton) [61,62]. We observed this difference also for our results obtained under natural marine conditions. Such discrepancy in biodegradation rates is further consistent with differences in the length of the cellulosic chains, the orientation of the fibers, their cross-section, their surface area, their pore structure, and the water retention level [62–64]. CLY fibers contain about 60% crystalline cellulose, with the remaining 40% being amorphous. In contrast, cotton includes 75–90% of crystalline and 10–25% of amorphous cellulose [65]. As a result, cotton has fewer regions prone to faster microbial degradation. In addition, cellulose chains in CV, CMD, and CLY are shorter with a low degree of polymerization (approximately 250 to 700 units) [65], while the cellulosic polymers within the cotton fibers have a high degree of polymerization (approximately 6,000 to 10,000 units). The lower crystallinity observed in CV and CMD results in higher moisture regain for regenerated cellulose [66]. This allows faster biodegradation than in natural cellulose fibers, whereas the latter has lower moisture regain due to the parallel configuration of cellulose I within the fiber structure [67,68]. Similar differences were observed elsewhere during the biodegradation of cellulose using a soil burial test, an activated sewage sludge test, and enzymatic hydrolysis [68]. Again, regenerated cellulosic fibers showed faster biodegradation. Overall, these parameters all play a role in the biodegradation rate. Still, other factors linked to the manufacturing process and the addition of various chemical compounds (dyes, protecting or repelling agents) can strongly and negatively impact the final degradation rate, even in the case of materials having in origin a more degradable structure, such as regenerated or natural cellulose [13,47].

In nature, *in situ* conditions such as solar light, seawater temperature, wind speed, salinity, nutrients such as nitrogen, microorganisms, and mechanical wave actions create a more or less favorable environment for biodegradation. The variability in such combined natural parameters influences each *in situ* experiment. This translates into a variability across the observed disintegration times that is difficult to interpret without the simplification achieved during tests in controlled settings. However, the Californian coastal waters, with an annual sea surface temperature between 13°C and 23°C, represent conditions comparable to that of several other temperate coastal environments across the globe [38]. In addition, our environmental observations agree with the bio-reactor results owing to a degradation time of about 28 days for cellulosic materials and close to no sign of disintegration for PLA, PET, and PP. This, importantly, was observed also for blend samples, where only the cellulosic portion degraded while the oil-based fibers remained intact. Overall, our results can be relevant to various geographical locations, and similar complementary approaches, using both tests in the environment and controlled settings, could help in the decisional process related to the mitigation of plastic pollution via alternative materials.

Since conventional oil-based fibers account for almost two-thirds of global fiber production [69,70] and 14.5% of plastic production by mass [1], some drastic measures are needed to transition to materials that allow biodegradation under natural conditions. Some cellulose-based materials are still leading the world’s market, but their contribution is negligible, with a share of 24% and 6% (2020) [71] for natural and regenerated cellulose, respectively. Linen, hemp, and jute are also cellulose fibers and account for 6% of the global market, while animal fibers (i.e., wool and silk) account for just over 1% of annual production [69]. While these replacements are known to cope better in nature, biodegradation processes can only be contemplated for virgin material, which is not often the case when consumer garments are considered. It is recognized that many different additives [72] are used in the textile industry, from nanomaterials integrated directly into textile substrates [73] to more durable water-repellent chemistry for textile finishing [74]. Each of these chemicals has specific functions including dyeing, protection from UV, antimicrobial, flame retardancy, oil, and water repellency. While these chemical processes are developed to optimize the quality of the materials, their application has a dramatic impact on the environment, prolonging or preventing the biodegradation of natural materials, thus promoting toxicity and detrimental effects on ecosystems [5,13,75].

Conclusively, although the so-called biodegradable PLA is opted for its potential to have a lesser impact on the environment at its end of life, such benefit over non-degradable conventional oil-based plastics is achieved only in its managed disposal. Combining environmental and bio-reactor degradation tests, we observed that in the lack of targeted management disposal, PLA may contribute to the problem of plastic pollution, as it cannot be associated with fast environmental biodegradation. Such observation agrees with previous investigations on PLA biodegradation [32,76]. A similar conclusion is obtained for cellulose/oil-plastic blend textiles, whereas their oil-plastic portion is recalcitrant to marine degradation. We stress, finally, how the use of misleading terminology could lead to detrimental consequences on the environment. Particularly the common practice of referring to industrially compostable materials as biodegradable plastic could propagate misconceptions about plastics that would degrade under any environmental conditions, misleading consumers and resulting in an increasing amount of plastic waste in the environment. To alleviate this misconception, we suggest that the term biodegradable should not be used for materials whose degradation has been tested in controlled settings only (e.g. industrial compost).

## Supporting information

S1 Fig Experimental setup for the sea surface and seafloor experiments at the Ellen Browning Scripps Memorial Pier located at Scripps Institution of Oceanography in La Jolla, California. (DOCX)

S2 Fig Experimental setup for the sea surface experiment at the Ellen Browning Scripps Memorial Pier located at Scripps Institution of Oceanography in La Jolla, California. A. Individual rectangles and squares for each material. B. Nylon mesh pockets to incubate the rectangles and squares for each material with their tag number in stainless steel. C. Cages in which the nylon pockets are kept. D. Final device submerged in sea surface waters.
(DOCX)

S3 Fig Experimental setup for the seafloor experiment at the Ellen Browning Scripps Memorial Pier located at Scripps Institution of Oceanography in La Jolla, California. A. Polyvinyl chloride (PVC) cage with a stainless-steel mesh. B. Soldered label on the bottom half of the PVC cage. C. Virgin material samples were inserted into the bottom half of the cages. D. Individual closed cages with zip ties attached. E. PVC cages apparatus set up securely positioned to both sides of the ropes. F. PVC cages settle on the seafloor at 10 m depth at Ellen Browning Scripps Memorial Pier located at Scripps Institution of Oceanography in La Jolla, California. G. Open PVC cages with material samples ready to be analyzed.
(DOCX)

S4 Fig Spectra using Raman Spectroscopy for regenerated cellulose material (Cellulose II -Lyocell (CLY)) and natural cellulose material (Cellulose I–organic virgin cotton (OCO)) at Day 0 (D0). (DOCX)

S5 Fig Time series in Julian days for 2019 and 2020 for the degradation experiments at the sea surface (light grey) and the seafloor levels (dark grey with dashed lines) at the Ellen Browning Scripps Memorial Pier located at Scripps Institution of Oceanography in La Jolla, California. Please note that only the cellulosic materials (Lyocell (CLY), Modal (CMD), Viscose (CV), organic virgin cotton (OCO), and non-organic virgin cotton (NOCO)) showed a complete degradation and hence the bio-based plastic, the blend, and the oil-based materials are not represented here. The numbers above each box correspond to the average temperature (°C) during this period. The symbol * corresponds to duplicate data given the same results for this period. NS signifies that there were no significant differences between the sea surface and seafloor treatments (p>0.05).
(DOCX)

S6 Fig Raman spectra at Day 0 and Day 84 for A. Polylactic acid (PLA); B. Blend sample comprises of Lyocell (CLY) and polyethylene terephthalate (PET) where only PET is represented and was the only representant remaining at Day 84; C. PET; D. Blend sample comprise of CLY and polypropylene (PP) where only PP is represented and was the only representant remaining at Day 84; E. PP. Note that variations in the peak’s relative intensities such as for instance between D and E are related to different fiber’s orientations under the laser and thus do not account for any structural change.
(DOCX)

S7 Fig Time series of selected meteorological data measured at the Ellen Browning Scripps Memorial Pier located at Scripps Institution of Oceanography in La Jolla, California during the experimental period for the sea surface and seafloor experiments from January 2019 to May 2020. A. Daily average for wind speed (m/s) and wave height (m). B. Daily average for seawater temperature (°C).
(DOCX)

S1 Table Vibrational assignment of the main observed Raman bands for cellulose-based fibers discriminated by their cellulose I (organic (OCO) and non-organic virgin cotton (NOCO)) and cellulose II (Lyocell (CLY), Modal (CMD) and Viscose (CV)) structure polylactic acid (PLA), polyethylene terephthalate (PET) and polypropylene (PP). Legend: ν, stretching; δ, bending; ρ, rocking; ω, wagging; τ, twisting; t, torsion; ϒ, out-of-plane vibration.
(DOCX)

S2 Table Bioreactor results showing the biodegradation based on CO_2_ production after 28 days of incubation for the following material types. A. Cellulose used as a reference, Lyocell (CLY), Modal (CMD), Viscose (CV), and organic virgin cotton (OCO). B Cellulose is used as a reference, polylactic acid (PLA), polyethylene terephthalate (PET), and polypropylene (PP). The experiments were conducted using duplicate samples.
(DOCX)

S1 Text Supplementary text for the extended version of the Raman microscopy method, the experimental design and the laboratory analysis. (DOCX)

S1 Graphical abstract (TIF)
