# Supplementary material for: Not so biodegradable: Polylactic acid and cellulose/plastic blend textiles lack fast biodegradation in marine waters
Source: PLoS One. 2023 May 24;18(5):e0284681. doi: 10.1371/journal.pone.0284681 (PMC10208507; doi:10.1371/journal.pone.0284681)
Supplement: S1 Fig — (DOCX) [file pone.0284681.s001.docx]

**SUPPLEMENTARY FIGURES**

**Figure S1:** Experimental setup for the sea surface and seafloor experiments at the Ellen Browning Scripps Memorial Pier located at Scripps Institution of Oceanography in La Jolla, California.
