# Supplementary material for: Not so biodegradable: Polylactic acid and cellulose/plastic blend textiles lack fast biodegradation in marine waters
Source: PLoS One. 2023 May 24;18(5):e0284681. doi: 10.1371/journal.pone.0284681 (PMC10208507; doi:10.1371/journal.pone.0284681)
Supplement: S2 Fig — A. Individual rectangles and squares for each material. B. Nylon mesh pockets to incubate the rectangles and squares for each material with their tag number in stainless steel. C. Cages in which the nylon pockets are kept. D. Final device submerged in sea surface waters. (DOCX) [file pone.0284681.s002.docx]

**SUPPLEMENTARY FIGURES**

**Figure S2:** Experimental setup for the sea surface experiment at the Ellen Browning Scripps Memorial Pier located at Scripps Institution of Oceanography in La Jolla, California. A. Individual rectangles and squares for each material. B. Nylon mesh pockets to incubate the rectangles and squares for each material with their tag number in stainless steel. C. Cages in which the nylon pockets are kept. D. Final device submerged in sea surface waters.
