# Supplementary material for: Not so biodegradable: Polylactic acid and cellulose/plastic blend textiles lack fast biodegradation in marine waters
Source: PLoS One. 2023 May 24;18(5):e0284681. doi: 10.1371/journal.pone.0284681 (PMC10208507; doi:10.1371/journal.pone.0284681)
Supplement: S3 Fig — A. Polyvinyl chloride (PVC) cage with a stainless-steel mesh. B. Soldered label on the bottom half of the PVC cage. C. Virgin material samples were inserted into the bottom half of the cages. D. Individual closed cages with zip ties attached. E. PVC cages apparatus set up securely positioned to both sides of the ropes. F. PVC cages settle on the seafloor at 10 m depth at Ellen Browning Scripps Memorial Pier located at Scripps Institution of Oceanography in La Jolla, California. G. Open PVC cages with material samples ready to be analyzed. (DOCX) [file pone.0284681.s003.docx]

**SUPPLEMENTARY FIGURES**

**Figure S3:** Experimental setup for the seafloor experiment at the Ellen Browning Scripps Memorial Pier located at Scripps Institution of Oceanography in La Jolla, California. A. Polyvinyl chloride (PVC) cage with a stainless-steel mesh. B. Soldered label on the bottom half of the PVC cage. C. Virgin material samples were inserted into the bottom half of the cages. D. Individual closed cages with zip ties attached. E. PVC cages apparatus set up securely positioned to both sides of the ropes. F. PVC cages settle on the seafloor at 10 m depth at Ellen Browning Scripps Memorial Pier located at Scripps Institution of Oceanography in La Jolla, California. G. Open PVC cages with material samples ready to be analyzed.
