# Supplementary material for: Not so biodegradable: Polylactic acid and cellulose/plastic blend textiles lack fast biodegradation in marine waters
Source: PLoS One. 2023 May 24;18(5):e0284681. doi: 10.1371/journal.pone.0284681 (PMC10208507; doi:10.1371/journal.pone.0284681)
Supplement: S4 Fig — (DOCX) [file pone.0284681.s004.docx]

**SUPPLEMENTARY FIGURES**


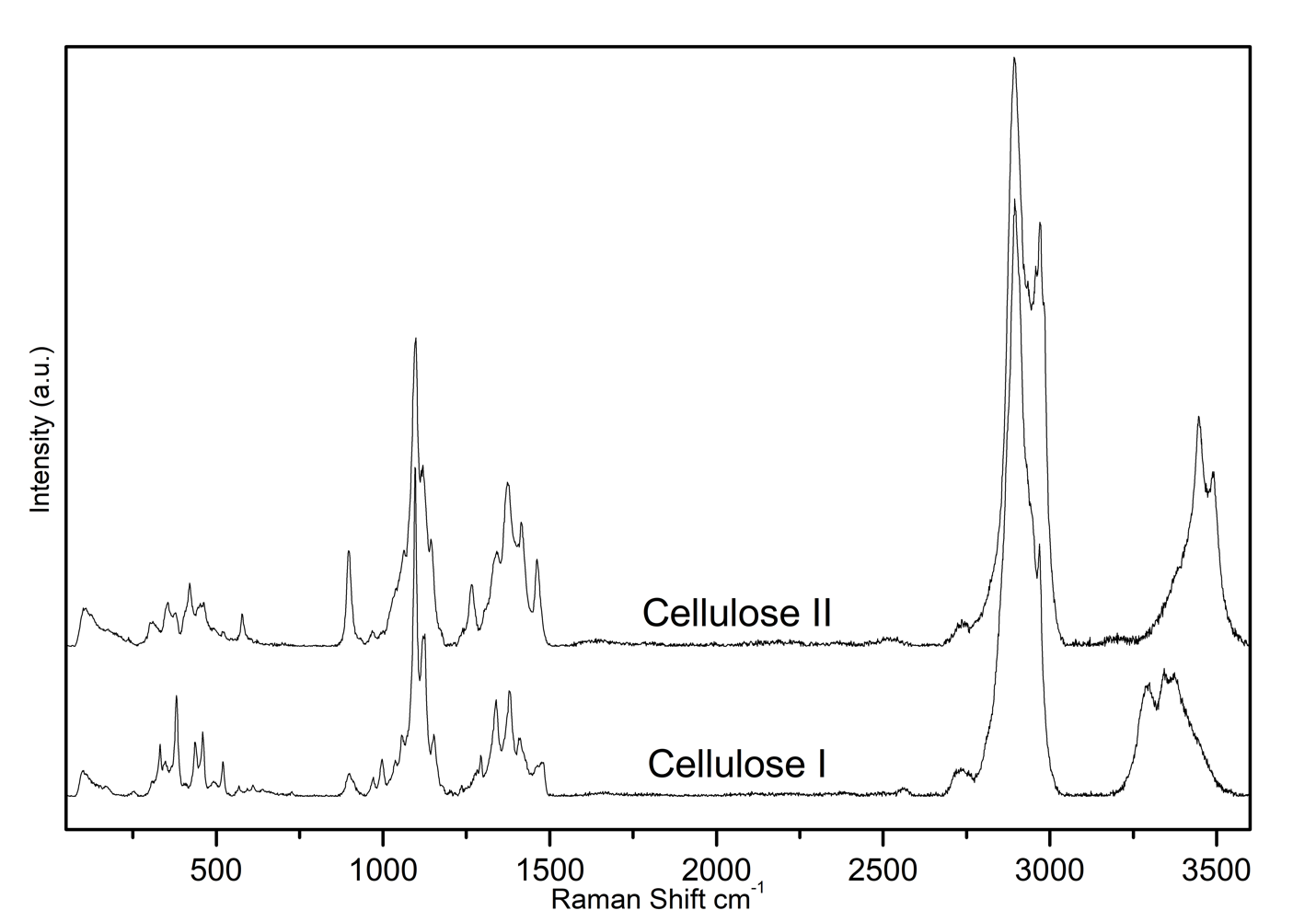


**Figure S4.** Spectra using Raman Spectroscopy for regenerated cellulose material (Cellulose II -Lyocell (CLY)) and natural cellulose material (Cellulose I – organic virgin cotton (OCO)) at Day 0 (D0).
