# Supplementary material for: Not so biodegradable: Polylactic acid and cellulose/plastic blend textiles lack fast biodegradation in marine waters
Source: PLoS One. 2023 May 24;18(5):e0284681. doi: 10.1371/journal.pone.0284681 (PMC10208507; doi:10.1371/journal.pone.0284681)
Supplement: S5 Fig — Please note that only the cellulosic materials (Lyocell (CLY), Modal (CMD), Viscose (CV), organic virgin cotton (OCO), and non-organic virgin cotton (NOCO)) showed a complete degradation and hence the bio-based plastic, the blend, and the oil-based materials are not represented here. The numbers above each box correspond to the average temperature (°C) during this period. The symbol * corresponds to duplicate data given the same results for this period. NS signifies that there were no significant differences between the sea surface and seafloor treatments (p>0.05). (DOCX) [file pone.0284681.s005.docx]

**SUPPLEMENTARY FIGURES**


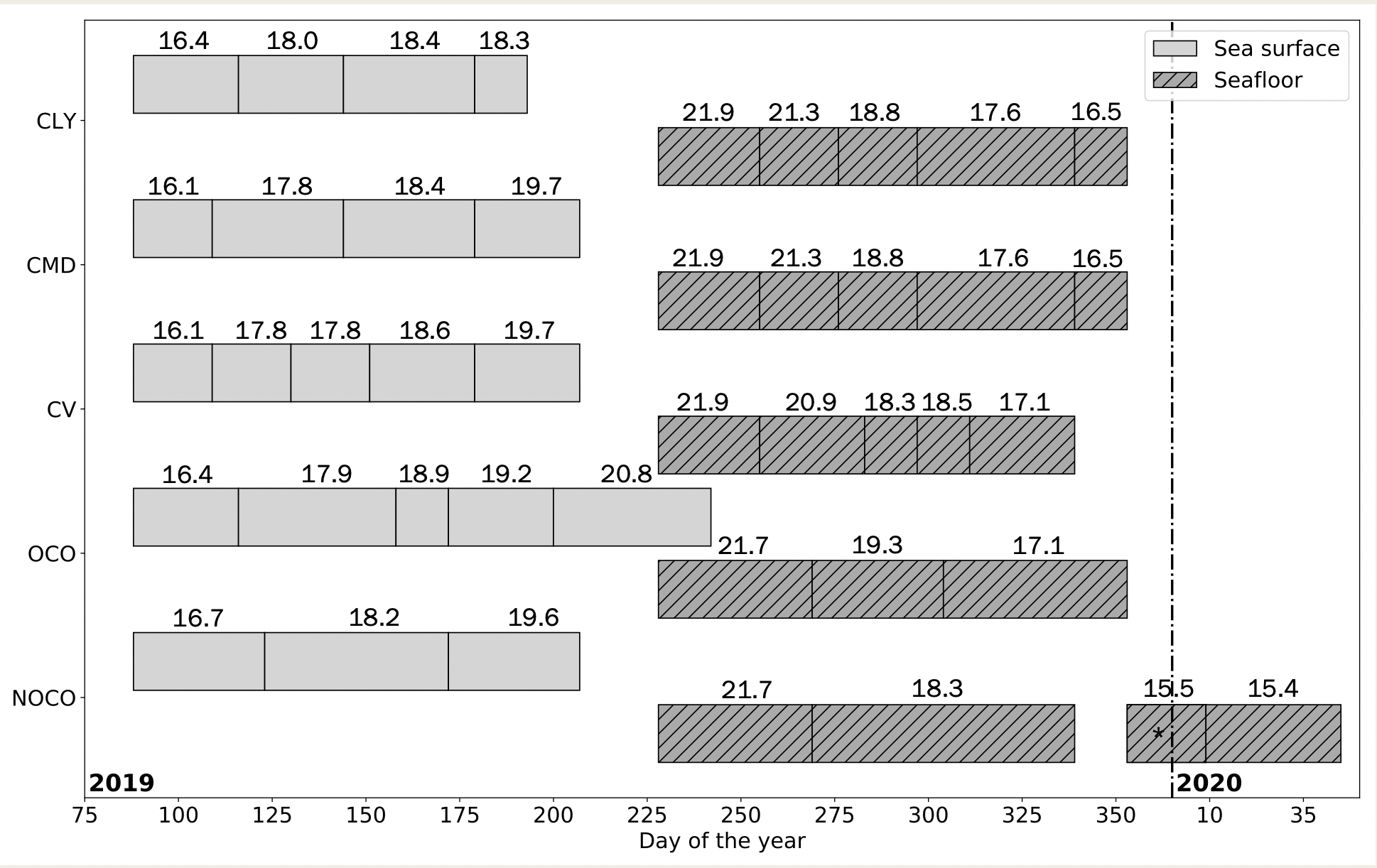


**Figure S5.** Time series in Julian days for 2019 and 2020 for the degradation experiments at the sea surface (light grey) and the seafloor levels (dark grey with dashed lines) at the Ellen Browning Scripps Memorial Pier located at Scripps Institution of Oceanography in La Jolla, California. Please note that only the cellulosic materials (Lyocell (CLY), Modal (CMD), Viscose (CV), organic virgin cotton (OCO), and non-organic virgin cotton (NOCO)) showed a complete degradation and hence the bio-based plastic, the blend, and the oil-based materials are not represented here. The numbers above each box correspond to the average temperature (^o^C) during this period. The symbol * corresponds to duplicate data given the same results for this period. NS signifies that there were no significant differences between the sea surface and seafloor treatments (p>0.05).
