# Supplementary material for: Not so biodegradable: Polylactic acid and cellulose/plastic blend textiles lack fast biodegradation in marine waters
Source: PLoS One. 2023 May 24;18(5):e0284681. doi: 10.1371/journal.pone.0284681 (PMC10208507; doi:10.1371/journal.pone.0284681)
Supplement: S6 Fig — Raman spectra at Day 0 and Day 84 for A. Polylactic acid (PLA); B. Blend sample comprises of Lyocell (CLY) and polyethylene terephthalate (PET) where only PET is represented and was the only representant remaining at Day 84; C. PET; D. Blend sample comprise of CLY and polypropylene (PP) where only PP is represented and was the only representant remaining at Day 84; E. PP. Note that variations in the peak’s relative intensities such as for instance between D and E are related to different fiber’s orientations under the laser and thus do not account for any structural change. (DOCX) [file pone.0284681.s006.docx]

**SUPPLEMENTARY FIGURES**


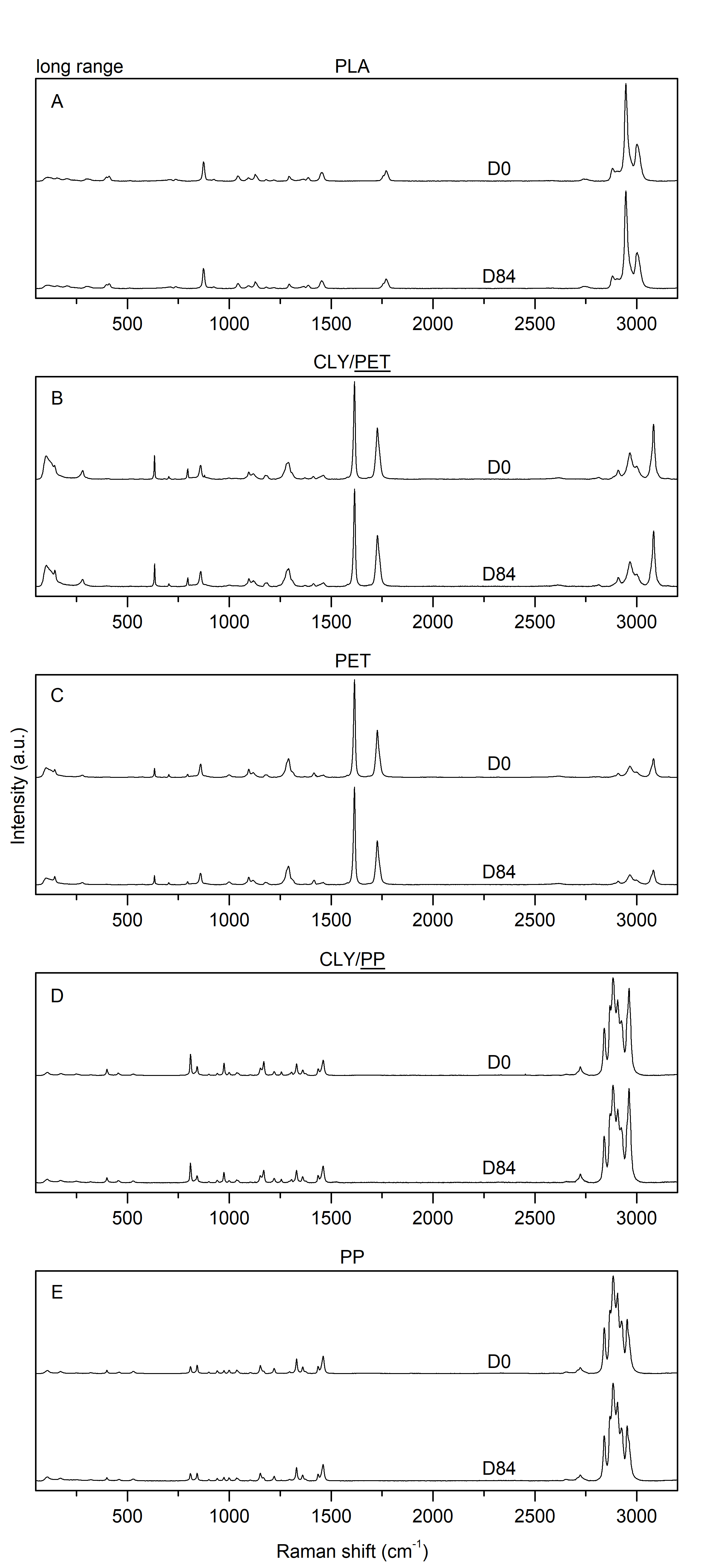


**Figure S6:** Raman spectra at Day 0 and Day 84 for A. Polylactic acid (PLA); B. Blend sample comprise of Lyocell (CLY) and polyethylene terephthalate (PET) where only PET is represented and was the only representant remaining at Day 84; C. PET; D. Blend sample comprises of CLY and polypropylene (PP) where only PP is represented and was the only representant remaining at Day 84; E. PP. Note that variations in the peak’s relative intensities such as between D and E are related to different fiber’s orientations under the laser and thus do not account for any structural change.
