# Supplementary material for: Not so biodegradable: Polylactic acid and cellulose/plastic blend textiles lack fast biodegradation in marine waters
Source: PLoS One. 2023 May 24;18(5):e0284681. doi: 10.1371/journal.pone.0284681 (PMC10208507; doi:10.1371/journal.pone.0284681)
Supplement: S7 Fig — A. Daily average for wind speed (m/s) and wave height (m). B. Daily average for seawater temperature (°C). (DOCX) [file pone.0284681.s007.docx]

**SUPPLEMENTARY FIGURES**


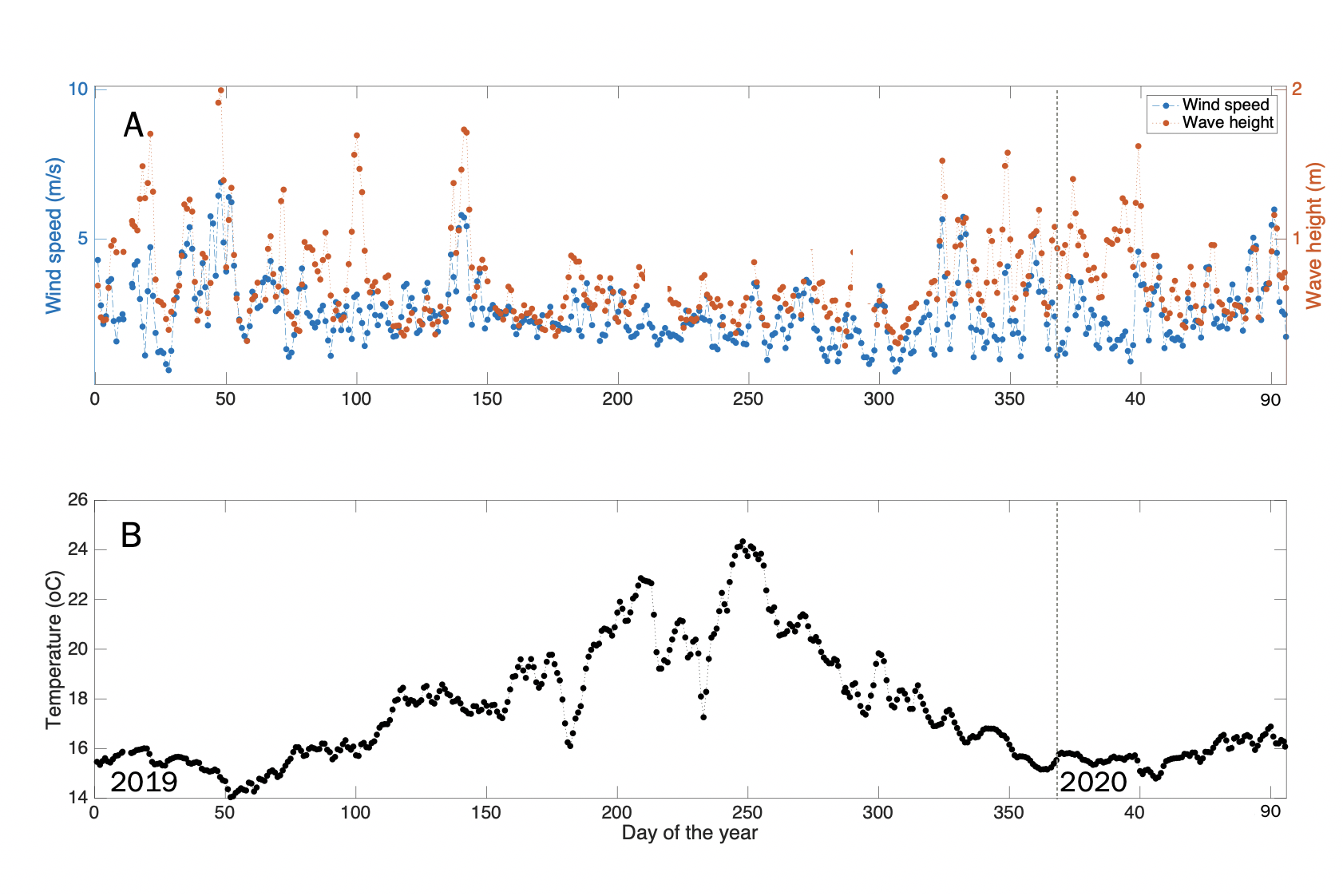


**Figure S7.** Time series of selected meteorological data measured at the Ellen Browning Scripps Memorial Pier located at Scripps Institution of Oceanography in La Jolla, California during the experimental period for the sea surface and seafloor experiments from January 2019 to May 2020. A. Daily average for wind speed (m/s) and wave height (m). B. Daily average for seawater temperature (^o^C).
