# Supplementary material for: Not so biodegradable: Polylactic acid and cellulose/plastic blend textiles lack fast biodegradation in marine waters
Source: PLoS One. 2023 May 24;18(5):e0284681. doi: 10.1371/journal.pone.0284681 (PMC10208507; doi:10.1371/journal.pone.0284681)
Supplement: S1 Table — Legend: ν, stretching; δ, bending; ρ, rocking; ω, wagging; τ, twisting; t, torsion; ϒ, out-of-plane vibration. (DOCX) [file pone.0284681.s008.docx]

**SUPPLEMENTARY TABLES**

**Table S1:** Vibrational assignment of the main observed Raman bands for cellulose-based fibers discriminated by their cellulose I (organic (OCO) and non-organic virgin cotton (NOCO)) and cellulose II (Lyocell (CLY), Modal (CMD) and Viscose (CV)) structure polylactic acid (PLA), polyethylene terephthalate (PET) and polypropylene (PP).

Legend: ν, stretching; δ, bending; ρ, rocking; ω, wagging; τ, twisting; t, torsion; ϒ, out-of-plane vibration.

| ^1^ Schenzel, K., & Fischer, S. (2001). NIR FT Raman spectroscopy–a rapid analytical tool for detecting the transformation of cellulose polymorphs. Cellulose, 8(1), 49-57. |
| --- |
| ^2^ Wiley, J. H., & Atalla, R. H. (1987). Band assignments in the Raman spectra of celluloses. |
| ^3^ - Kister, G., Cassanas, G., & Vert, M. (1998). Effects of morphology, conformation and configuration on the IR and Raman spectra of various poly (lactic acid) s. Polymer, 39(2), 267-273. |
| ^4^ - Bistričić, L., Borjanović, V., Leskovac, M., Mikac, L., McGuire, G. E., Shenderova, O., & Nunn, N. (2015). Raman spectra, thermal and mechanical properties of poly (ethylene terephthalate) carbon-based nanocomposite films. Journal of polymer research, 22(3), 39. |
| ^5^ - Lippert, T., Zimmermann, F., & Wokaun, A. (1993). Surface analysis of excimer-laser-treated polyethylene-terephthalate by surface-enhanced Raman scattering and x-ray photoelectron spectroscopy. Applied spectroscopy, 47(11), 1931-1942 |
| ^6^ - Boerio, F. J., S. K. Bahl, and G. E. McGraw. "Vibrational analysis of polyethylene terephthalate and its deuterated derivatives." Journal of Polymer Science: Polymer Physics Edition 14.6 (1976): 1029-1046. |
| ^7^ - Tashiro, K., Kobayashi, M., & Tadokoro, H. (1992). Vibrational spectra and theoretical three-dimensional elastic constants of isotactic polypropylene crystal: an important role of anharmonic vibrations.Polymer journal, 24(9), 899-916 |
| ^8^ - Andreassen, E. (1999). Infrared and Raman spectroscopy of polypropylene. In Polypropylene (pp. 320-328). Springer, Dordrecht. |
