# Supplementary material for: Not so biodegradable: Polylactic acid and cellulose/plastic blend textiles lack fast biodegradation in marine waters
Source: PLoS One. 2023 May 24;18(5):e0284681. doi: 10.1371/journal.pone.0284681 (PMC10208507; doi:10.1371/journal.pone.0284681)
Supplement: S2 Table — A. Cellulose used as a reference, Lyocell (CLY), Modal (CMD), Viscose (CV), and organic virgin cotton (OCO). B Cellulose is used as a reference, polylactic acid (PLA), polyethylene terephthalate (PET), and polypropylene (PP). The experiments were conducted using duplicate samples. (DOCX) [file pone.0284681.s009.docx]

**SUPPLEMENTARY TABLES**

**Table S2:** Bioreactor results showing the biodegradation based on CO_2_ production after 28 days of incubation for the following material types. A. Cellulose used as a reference, Lyocell (CLY), Modal (CMD), Viscose (CV), and organic virgin cotton (OCO). B Cellulose is used as a reference, polylactic acid (PLA), polyethylene terephthalate (PET), and polypropylene (PP). The experiments were conducted using duplicate samples.

A.

B.
