# Supplementary material for: Not so biodegradable: Polylactic acid and cellulose/plastic blend textiles lack fast biodegradation in marine waters
Source: PLoS One. 2023 May 24;18(5):e0284681. doi: 10.1371/journal.pone.0284681 (PMC10208507; doi:10.1371/journal.pone.0284681)
Supplement: S1 Text — (DOCX) [file pone.0284681.s010.docx]

Supplementary Materials for

**Not so biodegradable: polylactic acid and cellulose/plastic blend textiles lack fast biodegradation in marine waters**

Sarah-Jeanne Royer*, Francesco Greco, Michaela Kogler, Dimitri, D., Deheyn

*Corresponding author. Email: [sroyer@ucsd.edu](mailto:sroyer@ucsd.edu)

Text S1: Supplementary text for the extended version of the *Raman Microscopy* method, the *Experimental design* and the *Laboratory analysis.*

*Raman microscopy* (extended version)

Confocal Raman spectroscopy on untreated micro-fibres was carried out at the State Key Laboratory of Continental Dynamics, Department of Geology, Northwest University of Xi’an, using an In Via Renishaw Confocal Laser Raman microscope equipped with an automated sample stage. Measurements were obtained at room temperature using a 514.5 nm wavelength Ar^+^ laser source. Spectra were collected in the Stokes Raman shift with a short- and long-range up to 3800 cm^-1^ using 1800 lines/mm and 600 lines/mm gratings and a cooled CCD sensor for signal detection. The spectrometer was calibrated with a silicon chip standard before each experimental session and re-calibration was performed every few hours during day-long experiments. For micro-fibres identification and structural characterization punctual data, laser spot size of ca. 1-2 µm, were collected using 50X, 100X objectives (N.A. = 0.75; 0.85) on a Leica DM LM microscope and a laser power at the sample surface of 0.5-5 mW for 20-120 s of total acquisition time. The laser power and spectral acquisition time were carefully chosen experimentally at each sample to obtain the best possible spectral quality, higher signal/noise ratio, and lower fluorescence intensity, and to avoid laser-induced thermal alteration of micro-fibres which, when observed, resulted in a progressive polymers carbonization, as confirmed by the appearance of amorphous carbon D and G peaks, and local burning of the micro-fibres. Laser power at the sample surface was monitored with a Thorlabs PM100D Energy Meter Console equipped with a S120C Si-Photodiode Power Sensor. A power of 3 mW at the sample surface was the most frequently used for both cellulosic and non-cellulosic samples allowing better spectral quality, comparison, and measurements reproducibility.

A total of 501 recorded spectra was considered during this investigation. The study was divided into two major steps: 1) An identification stage to confirm the identity of the virgin materials (at D0) through the literature-based, assignment of the spectral bands to the involved molecular vibrations. This was followed by an assessment of the within-sample spectral heterogeneity (differences between spectra collected on the individual micro-fibres) at the virgin materials (D0) to recognize and exclude from further structural comparison the variability caused by anisotropic effects under Raman excitation or other structural changes intrinsic to the virgin samples. 2) The investigation of structural alteration in degrading materials through the comparison of their spectra with the within-sample heterogeneity observed at D0. Particularly Polylactic Acid (PLA), polyethylene terephthalate (PET), Polypropylene (PP), and blend materials (Lyocell (CLY)/PET and CLY/PP) were analysed at D84 while cellulose-based materials were analysed at their last two sampling steps of a complete degradation cycle, that is D21 and D28 for CLY, D7 and D14 for CMD and CV, D21 and D28 for OCO and D28 and D35 for NOCO. In reason of their strong original and growing fluorescence during degradation cellulose-based materials were analysed in their raw spectral form and except for regenerated cellulose fibres (CLY, CMD, and CV) at D0, whereas in such samples good spectral quality allowed the calculation of some peaks ratios on background processed spectra, no further background removal or spectral processing was performed. On the other hand, spectra on bio-based plastic and oil-based materials as well as the oil-based component of the blend samples, resulting in an excellent profiles quality, have been processed for background subtraction and curve fitting (a combination of Lorentzian, Gaussian, and Voigt models) using Crystal Sleuth and OriginPro 8.5 software. Obtained parameters (peak intensity I, integrated area A, full width at half maximum FWHM) and calculated ratios have been used to assess and compare, through paired t-tests on groups size of 10 pairs (OriginPro 8.5), the degree of crystallinity, of polymerization and in general the structural heterogeneity between samples at D0 and D84 to test the hypothesis of structural degradation.

Spectra were collected using the following wavenumber ranges: full range from 20 up to 3800-4000 cm^-1^, first-order region up to 1600-2000 cm^-1^, and second-order region from 2700 cm^-1^ up to 3800-4000 cm^-1^. In the case of high fluorescence background, new spectra would be recorded for a minimum of 12 spectra per different types of material to ensure good replicability of every sample. In the case of possible photothermal damage due to laser illumination, new spectra would also be recorded to ensure the good quality of the measurements.

*Experimental design* (extended version)

*Sea surface*: The upper side of the larger pocket was folded and stapled four times to prevent any loss of material and zip-tied twice as an extra safety. The individual pockets were randomly placed in secured metallic cages to overcome the rough oceanic conditions (Supplementary Fig. 7C). A total of four cages containing the samples were attached to a polypropylene rope of 2.5 cm diameter and 2 x 2.26 kg weights were tied to each extremity of the ropes followed by two yellow buoys to keep all the cages at the same level horizontally. The cages were also attached to a thinner yellow polypropylene rope as safety to avoid any loss of the samples or in case the thicker rope would get entangled or broken (Supplementary Fig. 6 and 7D). Cages were pulled out of the water weekly and the Nylon pockets with their respective samples were taken to the laboratory for analysis.

*Seafloor*: The bottom half of each cage was engraved, using a soldering iron, to correlate them with their sample number (Supplementary Fig. 8B). Using a screw gun, four holes were drilled into the top half of the cages where two zip ties were inserted adjacent to each other. A rectangular and a square for each material type were placed per cage (Supplementary Fig. 8C) before the top was securely screwed on (Supplementary Fig. 8D). The cages were then tied using zip ties vertically along two polypropylene ropes of 2.5 cm diameter 15 cm apart with a 2 x 2.26 kg weight on each extremity of the ropes (Supplementary Fig. 8E). After the zip ties were secured, the excess zip tie length was cut with scissors and the sharp edges were melted using a soldering iron. All ropes that were cut and tied were burned and melted together, using the soldering iron, to decrease unraveling while exposed to the seafloor elements. To avoid entanglement the apparatus was folded in half into an “S” figure before being lowered into the ocean (Supplementary Fig. 8F). An additional yellow polypropylene rope was used as a backup to secure the apparatus and tied to the adjacent pillar for safety. The cages were pulled out of the water weekly (Supplementary Fig. 8G) and each bottom half of the cage was removed containing the material, screwed with a PVC cap, and gently dunked ten times in a bucket of seawater to rinse the sediment off the samples within each cage. The samples were then taken to the laboratory for analysis.

*Laboratory analysis* (extended version)

Sea surface and seafloor samples were analyzed using the same protocol. After their collection at the pier, the rectangle, and square samples were gently removed and untangled using tweezers and a flat steel spatula. The square was placed over the side of each label while the rectangle with the right indentation positioned on the upper right corner was placed on a labeled blackboard with their identifier for photographs. Images were taken using a professional photo studio box with a light-emitting diode and photographs were taken using a single-lens reflex camera Nikon 12-megapixel camera. A subsample of the individual squares (a square of 1 cm^2^) was collected and dried for analysis under scanning electron microscopy. The remaining samples were put back in the water.

SUPPLEMENTARY REFERENCES

1. Schenzel, K. & Fischer, S. NIR FT Raman spectroscopy - A rapid analytical tool for detecting the transformation of cellulose polymorphs. *Cellulose* **8**, 49–57 (2001).

2. Wiley, J. H. & Atalla, R. H. the Institute of Paper Chemistry , Appleton , Wisconsin Ipc Technical Paper Series Number 226 Raman Spectra of Celluloses James H . Wiley and Rajai H . Atalla. (1987).

3. Kister, G., Cassanas, G. & Vert, M. Effects of morphology, conformation and configuration on the IR and Raman spectra of various poly(lactic acid)s. *Polymer (Guildf).* **39**, 267–273 (1998).

4. Bistričić, L. *et al.* Raman spectra, thermal and mechanical properties of poly(ethylene terephthalate) carbon-based nanocomposite films. *J. Polym. Res.* **22**, (2015).

5. Lippert, T. H., Zimmermann, F. & Wokaun, A. Surface analysis of excimer-laser-treated polyethylene-terephthalate by surface-enhanced raman scattering and X-ray photoelectron spectroscopy. *Appl. Spectrosc.* **47**, 1931–1942 (1993).

6. Boerio, F. J. & Bahl, S. K. Vibrational analysis of polyethylene terephthalate and its deuterated derivatives. *J. Polym. Sci.* **14**, 1029–1046 (1976).

7. Tashiro, K., Kobayashi, M. & Tadokoro, H. Vibrational spectra and theoretical three-dimensional elastic constants of isotactic polypropylene crystal: An important role of anharmonic vibrations. *Polym. J.* **24**, 899–916 (1992).

8. Andreassen, E. Infrared and Raman spectroscopy of polypropylene. 320–328 (1999) doi:10.1007/978-94-011-4421-6_46.
